# Supplementary material for: Ameliorative Effects of Aspergillus awamori against the Initiation of Hepatocarcinogenesis Induced by Diethylnitrosamine in a Rat Model: Regulation of Cyp19 and p53 Gene Expression
Source: Antioxidants (Basel). 2021 Jun 7;10(6):922. doi: 10.3390/antiox10060922 (PMC8228954; doi:10.3390/antiox10060922)
Supplement: Supplementary file 1 [file antioxidants-10-00922-s001.zip › antioxidants-1225107-supplementary.pdf]

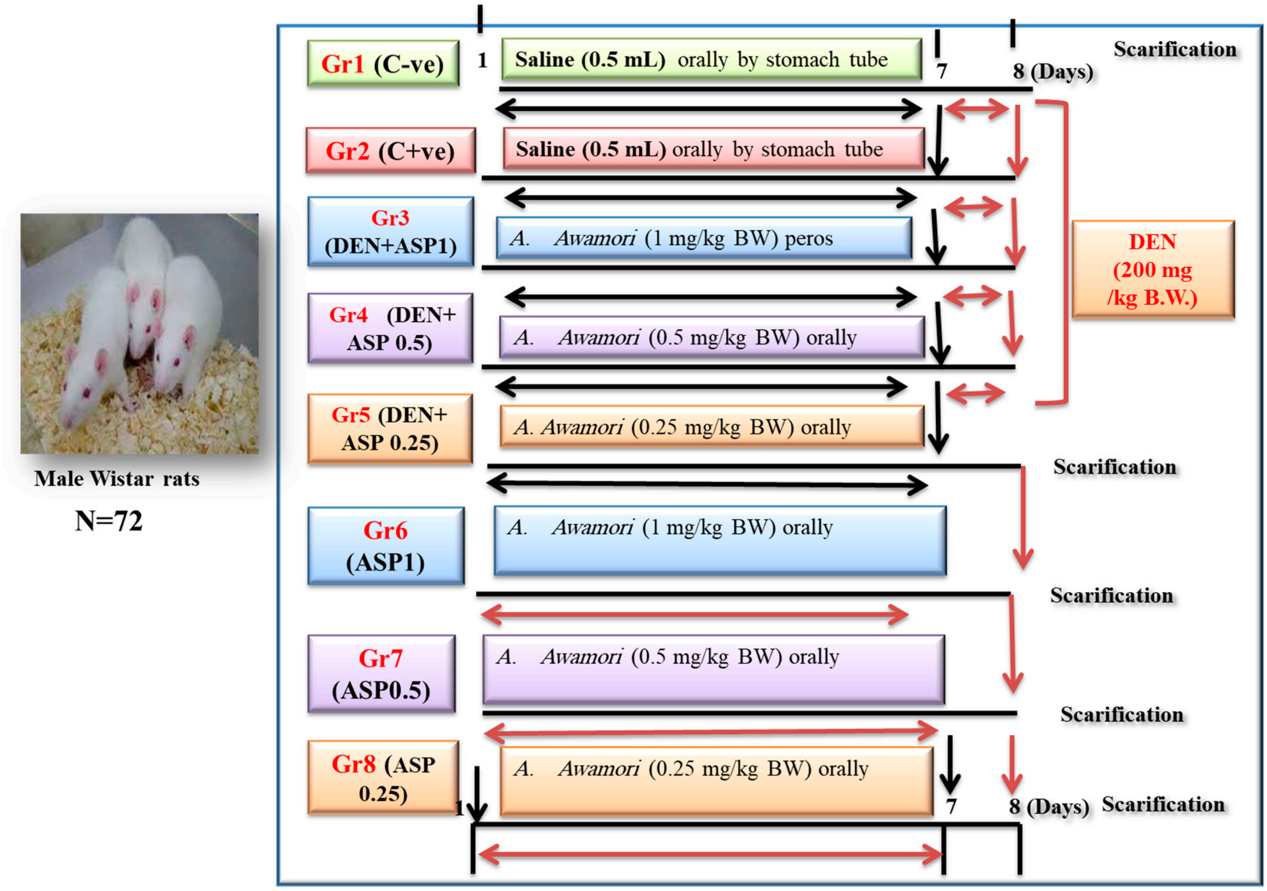

Supplementary Figure S1. Experimental design

Supplementary Table S1. Ingredients composition of the regular diet

**Feed ingredients**	**%**
wheat flour	22.5
corn starch	27
essential fatty acids	0.6
soybean powder	20
sucrose	10
Cellulose	2.6
corn oil	5
Vitamins*	2
Minerals**	10

*Vitamins contained (A 0.6 mg/kg, D 1000IU/kg, E 35 mg/kg, niacin 20 mg/kg, pantothenic acid 8 mg/kg, riboflavin 0.8 mg/1000 kcal, thiamin 4 mg/kg, B6 50 µg/kg and B12 7 mg/kg of diet).

**Minerals contained (calcium 5 g/kg, Phosphorus 4 g/kg, fluoride 1 mg/kg, iodine 0.15 mg/kg, chloride 5 mg/kg, iron 35 mg/kg, copper 5 mg/kg, magnesium 800 mg/kg, potassium 35 mg/kg, manganese 50 mg/kg and sulfur3 mg/kg of diet).

Supplementary file 2

UPLC-PDA-MS/MS for metabolite analysis

Detailed description of bioactive compounds found in *A. awamori* in our study

Compound 1

Exhibited an $[M-H]^-$ ion at *m/z* 195 and MS/MS fragment ions at *m/z* 177, 159, 129, 99, and 75, which are characteristic for gluconic acid [**1-**4].

Compounds 2, 3, 4

Demonstrated an $[M-H]^-$ ion at *m/z* 191 with different retention times. Compounds **2** and **4** showed the same MS/MS fragmentation profile at *m/z* 129, 111, 87, 85. The fragment ion at *m/z* 111 is corresponding to $[M-H-CO_2-2H_2O]^-$. Compound **3** has the same fragmentation pattern with an additional fragment at *m/z* 155 due to the neutral loss of two water molecules. Comparing with the literature, compounds **2** and **4** were identified as citric acid and citric acid isomer, while compound **3** as isocitric acid [**5**].

Compound 5

Showed an $[M-H]^-$ ion at *m/z* 205 and fragment ions at *m/z* 143, 111, 87 in the MS/MS spectrum. The fragment ion at *m/z* 111 indicated a citric acid derivative, and the fragment at *m/z* 143 implied a methyl substitution compared with the fragment 129 of citric acid. Compared with the literature, compound **5** was tentatively identified as a methyl derivative of citric acid.

Compound 6

Exhibited an $[M-H]^-$ ion at *m/z* 147 and fragment ions at *m/z* 147 and 103 in the MS/MS spectrum. These fragments are characteristic of cinnamic acid **[6].**

Compound 7

Demonstrated an $[M-H]^-$ ion at *m/z* 161 and MS/MS fragment ions at *m/z* 129 and 101, which matches methyl salicylate in databases.

Compounds 8 and 10

Showed an $[M-H]^-$ ion at *m/z* 219 and share the MS/MS fragment ions at *m/z* 157 and 143, 111, and 87. The fragments at *m/z* 111 and 87 indicated citric acid derivatives. The fragment ions at *m/z* 143 and 157 imply two methyl

substituents compared with compounds **4** and **5**. By searching in the databases, compound **8** was identified as 1,3-dimethyl citrate and compound **10** as 1,5-dimethyl citrate.

Compound 9

Demonstrated an $[M-H]^-$ ion at *m/z* 153 and MS/MS fragment ion at *m/z* 109 and 108, which matches the data for gentisic acid in databases.

Compound 11

Exhibited an $[M-H]^-$ ion at *m/z* 175 and MS/MS fragment ions at *m/z* 157, 129, 115, 113, 85, which is in concordance with ascorbic acid analytical data **[7].**

Compound 12

With an $[M-H]^-$ ion at *m/z* 563 and fragment ions at *m/z* 545, 503, 473, 443, 425, 412, 406, 383, 365, 353, 311, 378, 293, 233 in MS/MS spectrum was detected. The fragment ion at *m/z* 473 is corresponding to $(M - H\text{-}120)^-$, and 383 is corresponding to $(M\text{-}120\text{-}90)^-$. These fragment ions ($[M - H\text{-}120)^-$, $[M\text{-}120\text{-}90^-]$) are characteristic for flavone glycosides with two *C*-glucosides in positions 6 and 8. The absorption maxima of this compound were detected by PDA and found as 273 and 322 nm. Compound **12** was identified from the published literature as apigenin-6,8-C-diglucoside (vicenin II) [**8,9**].

Compound 27

Is another flavonoid identified. It exhibited an $[M-H]^-$ ion at *m/z* 287 and fragmentation ions *m/z* 287, 269 $[M\text{-}H\text{-}H_2O]^-$, 251 $[M\text{-}H\text{-}2H_2O]^-$, 225, 201, 155, 125. These fragments indicated that compound **27** has the skeleton of dihydroflavonol with the absence of the hydroxyl group at the position C-3′ of the B ring **[10]** and was identified as aromadendrin.

Compound 14

Showed an $[M-H]^-$ ion at *m/z* at 415 with MS/MS fragment ions at *m/z* 191, 175, 139, 119, 101, 89. The fragment ion at 175 *m/z* is matching a dihydroxy coumarin derivative, 191 matches [M-glucosyl(162)-carboxyl(44)-H_2O(18)]. Tentatively, this compound was identified as tetrahydroxy coumarin-3́-carboxylic acid-β-D-glucoside derivative.

Compound 18

Is another coumarin identified as isofraxidin with an $[M-H]^-$ ion at *m/z* 221 and fragment ions at *m/z* 206, 162, 150, 133.

Compound 23

Showed an $[M-H]^-$ ion at *m/z* at 413 (two Da less than compound **14**) with MS/MS fragment ions at *m/z* 252, 234, 193, 177, 175, 163, 162, 134, 119. The fragment ions at *m/z* 177 and 175 suggested dihydroxy dihydrocoumarin derivative, *m/z* 252 is corresponding to $[M-H-162]^-$, and *m/z* 193 is corresponding to $[M-H-162-59]^-$ that indicated the presence of glucose and carboxymethyl groups. Tentatively, this compound was identified as methyl-dihydroxy-dihydrocoumarin-3́-carboxymethyl-β-D-glucoside.

Compounds 13 and 16

With $[M-H]^-$ ion at *m/z* 163 and 161, and MS/MS fragment ions at 121 and 119, respectively, were identified as phthalic acid and *p*-coumaric acid. While the $[M-H]^-$ ion at *m/z* 151 for compound **15** with MS/MS fragment ion at *m/z* 107 proposed anisic acid.

Compound 17

With an $[M-H]^-$ ion at *m/z* 187 showed the MS/MS fragment ions at *m/z* 169, 143, 125, 123, 97. The fragment ion at *m/z* 125 corresponding to $[M-H-44-18]^-$, *m/z* 169 corresponding to $[M-H18]^-$, and 143 corresponding to $[M-H-44]^-$ are characteristic for gallic acid and benzoic acid derivatives [**11,**6].

Compounds 20, 22, 25(a, b), 28, 29 (a, b), 30-36, and 38

Were identified as fatty acid derivatives of octadecenoic, octadecadienoic and octadecanoic acids based on their corresponding $[M-H]^-$ and MS/MS fragment ions.

***Compounds 22 and 25* (a, b)**

Exhibited an $[M-H]^-$ at *m/z* 329. MS/MS fragment ions for compound 22 are 314, 299, 271, 229, 211, 171, 157, 139, 127, 99, which matches those for trihydroxy octadecenoic acid [**12,**13]. Compounds 25 (a, b) were identified as an isomer of 22.

Compound 20

Has 2 Da extra to compound 22 with a similar fragmentation pattern and additional fragment ions at *m/z* 295 and 201. It was suggested to be a trihydroxy octadecanoic acid derivative.

Compound 28

Had an $[M-H]^-$ at *m/z* 311. Its MS/MS fragments at *m/z* 223, 183 match those for 15,16-dihydroxy-9,12-octadecadienoic acid [**14**].

Compounds **29** (a, b) demonstrated an $[M-H]^-$ at *m/z* 313. Other fragments are 277, 201, 171, 165, 155, 127 suggested hydroperoxyl octadecenoic acid isomers [**13**].

Compound 38

Has an $[M-H]^-$ at *m/z* 297 and gave the fragment ion 279 and 251 in MS/MS spectrum, which is characteristic for the epoxy derivative of octadecanoic acid (molecular weight 284).

Compounds 31 (a, b)

With an $[M-H]^-$ at *m/z* 315 had 32 Da extra than octadecanoic acid. Its fragmentation showed the ions at *m/z* 297, 279 corresponding to the loss of two water molecules, which supports the identification as dihydroxy octadecanoic acid isomers.

Compounds 32 (a, b) and 35 (a, b)

Have an extra 42 Da as compared with compounds 28 and 31, respectively. The MS/MS fragments of these compounds showed fragment ions at *m/z* 313 and 315, respectively corresponding to $[M-43]^-$suggesting a propyl group. The other fragments matched compounds **28** and **31**. Based on this fragmentation pattern, compounds **32** and **35** were tentatively identified as propyl esters and their isomers of compounds **28** and **31.**

Compound 33 and 34

Showed an $[M-H]^-$ at *m/z* 295. Compound **33** demonstrated MS/MS fragment ions at *m/z* 277, 195, 171, 113, which is in good concordance with the data of 13(S)-hydroxy octadecadienoic acid (α-artemisolic acid) [**15**]. Compound 34 was identified as an isomer of **33.**

Compound 39

With an $[M-H]^-$ at *m/z* 271 showed fragment ions at *m/z* 271, 253 $[M-H-H_2O]^-$ and 225. These data suggested hydroxy palmitic acid. The fragment *m/z* 225 was found at the base peak in the MS/MS spectrum, which is in concordance with 2-hydroxy palmitic acid [**16**].

Compounds 26 and 30

Were identified as ursolic acid derivatives as compared with literature of **Abdelaziz et al. [17]**.

Compound 24

Demonstrated an [M-H]$^-$ at *m/z* 1085 with fragment ion at *m/z* 865 as the base peak. This fragment indicated that compound **24** is a derivative of procyanidin C1 [**18**]. This compound indicated that *A. awamori* contains tannins of condensed type.

This assortment of such bioactive compounds can explain the exhibited biological potential of *A. awamori* in our study. Among these identified constituents, citric acid and its isomers, derivatives of unsaturated fatty acids such as octadecenoic and octadecadienoic acids, and derivatives of saturated fatty acids as stearic (octadecanoic acid) and palmitic acids which are the foremost components.

References

[1] Liu XR, Zheng XF, Ji SZ, et al. (2010) Metabolomic analysis of thermally injured and/or septic rats. Burns 36:992–998. https://doi.org/10.1016/j.burns.2010.03.015

[2] Deng J and Yang Y (2013) Chemical fingerprint analysis for quality assessment and control of Bansha herbal tea using paper spray mass spectrometry. Anal Chim Acta 785:82–90. https://doi.org/10.1016/j.aca.2013.04.056

[3] Yang H, Lin W, Zhang J, et al. (2014) Metabonomic analysis of the toxic effects of TM208 in rat urine by HPLC-ESI-IT-TOF/MS. J Chromatogr B Anal Technol Biomed Life Sci 959:49–54. https://doi.org/10.1016/j.jchromb.2014.03.036

[4] Felipe DF, Brambilla LZS, Porto C, et al. (2014) Phytochemical analysis of Pfaffia glomerata inflorescences by LC-ESI-MS/MS. Molecules 19:15720–15734. https://doi.org/10.3390/molecules191015720

[5] Al Kadhi O, Melchini A, Mithen R, Saha S. (2017) Development of a LC-MS/MS Method for the Simultaneous Detection of Tricarboxylic Acid Cycle Intermediates in a Range of Biological Matrices. J Anal Methods Chem 2017:1–12. https://doi.org/10.1155/2017/5391832

[6] Basu S, Patel VB, Jana S, Patel H (2013) Liquid chromatography tandem mass spectrometry method (LC-MS/MS) for simultaneous determination of piperine, cinnamic acid and gallic acid in rat plasma using a polarity switch technique. Anal Methods 5:967–976. https://doi.org/10.1039/c2ay26289d

[7] Szultka M, Buszewska-Forajta M, Kaliszan R, Buszewski B (2014). Determination of ascorbic acid and its degradation products by high-performance

liquid chromatography-triple quadrupole mass spectrometry. Electrophoresis 35:585–592. https://doi.org/10.1002/elps.201300439.

[8] Barreca D, Bellocco E, Caristi C, et al. (2011) Flavonoid profile and radical-scavenging activity of Mediterranean sweet lemon (Citrus limetta Risso) juice. Food Chem 129:417–422. https://doi.org/10.1016/j.foodchem.2011.04.093

[9] Ibrahima RM, El-Halawany AM, Saleh DO, et al. (2015) HPLC-DAD-MS/MS profiling of phenolics from securigera securidaca flowers and its anti-hyperglycemic and anti-hyperlipidemic activities. Rev Bras Farmacogn 25:134–141. https://doi.org/10.1016/j.bjp.2015.02.008

[10] Chen G, Li X, Saleri F, Guo M (2016) Analysis of Flavonoids in Rhamnus davurica and Its Antiproliferative Activities. Molecules 21:1275. https://doi.org/10.3390/molecules21101275

[11] Kim H, Roh H, Lee HJ, et al. (2003) Determination of phloroglucinol in human plasma by high-performance liquid chromatography-mass spectrometry. J Chromatogr B Anal Technol Biomed Life Sci 792:307–312. https://doi.org/10.1016/S1570-0232(03)00316-7

[12] Llorent-Martinez EJ, Spinola V, Gouveia S, Castilho PC (2015) HPLC-ESI-MSn characterization of phenolic compounds, terpenoid saponins, and other minor compounds in Bituminaria bituminosa. Ind Crops Prod 69:80–90. https://doi.org/10.1016/j.indcrop.2015.02.014

[13] Agalar HG, Ciftci GA, Goger F, Kırımer N (2018) Activity guided fractionation of Arum italicum miller tubers and the LC/MS-MS profiles. Rec Nat Prod 12:64–75. https://doi.org/10.25135/rnp.06.17.05.089

[14] Yang N-Y, Yang Y-F, Li K (2013) Analysis of Hydroxy Fatty Acids from the Pollen of Brassica campestris L. var. oleifera DC. by UPLC-MS/MS. J Pharm 2013:1–6. https://doi.org/10.1155/2013/874875

[15] Abu-Reidah IM, Ali-Shtayeh MS, Jamous RM, et al. (2015) Comprehensive metabolite profiling of Arum palaestinum (Araceae) leaves by using liquid chromatography-tandem mass spectrometry. Food Res Int 70:74–86. https://doi.org/10.1016/j.foodres.2015.01.023

[16] Kokotou MG, Mantzourani C, Bourboula A, et al. (2020) A Liquid Chromatography-High Resolution Mass Spectrometry (LC-HRMS) Method for the Determination of Free Hydroxy Fatty Acids in Cow and Goat Milk. Molecules

25:3947. https://doi.org/10.3390/molecules25173947

[17] Abdelaziz S, Hassan WHB, Elhassanny AEM, et al. (2020) Ultra performance liquid chromatography-tandem mass spectrometeric analysis of ethyl acetate fraction from saudi Lavandula coronopifolia Poir and evaluation of its cytotoxic and antioxidant activities. J HerbMed Pharmacol 9:268–276. https://doi.org/10.34172/jhp.2020.34

[18] Chang Z, Zhang Q, Liang W, et al. (2019) A Comprehensive Review of the Structure Elucidation of Tannins from Terminalia Linn. Evidence-Based Complement Altern Med 2019:1–26. https://doi.org/10.1155/2019/8623909
